# In search of quality evidence for lifestyle management and glycemic control in children and adolescents with type 2 diabetes: A systematic review

**DOI:** 10.1186/1471-2431-10-97

**Published:** 2010-12-23

**Authors:** Steven T Johnson, Amanda S Newton, Meera Chopra, Jeanette Buckingham, Terry TK Huang, Paul W Franks, Mary M Jetha, Geoff DC Ball

**Affiliations:** 1 Centre for Nursing & Health Studies, Faculty of Health Disciplines, 1 University Drive Athabasca University, Athabasca, AB, Canada; 2 Department of Pediatrics, University of Alberta, 8213 Aberhart Centre, 11402 University Avenue, Edmonton, AB, Canada, T6G 2P3; 3 Mount Sinai Hospital, Joseph and Wolf Lebovic Health Complex, 60 Murray Street, 4th Floor, Toronto, Ontario, Canada, M5T 3L9; 4 John W. Scott Health Sciences Library, University of Alberta, WMC 2K4.01, Edmonton, AB Canada T6G 2R7; 5 Eunice Kennedy Shriver National Institute of Child Health and Human Development, Bethesda, MD, USA; 6 Genetic Epidemiology and Clinical Research Group, Department of Public Health and Clinical Medicine, Division of Medicine, Umeå University Hospital, Umeå, Sweden

## Abstract

**Background:**

Our purpose was to evaluate the impact of lifestyle behavior modification on glycemic control among children and youth with clinically defined Type 2 Diabetes (T2D).

**Methods:**

We conducted a systematic review of studies (randomized trials, quasi-experimental studies) evaluating lifestyle (diet and/or physical activity) modification and glycemic control (HbA1c). Our data sources included bibliographic databases (EMBASE, CINAHL^®^, Cochrane Library, Medline^®^, PASCAL, PsycINFO^®^, and Sociological Abstracts), manual reference search, and contact with study authors. Two reviewers independently selected studies that included any intervention targeting diet and/or physical activity alone or in combination as a means to reduce HbA1c in children and youth under the age of 18 with T2D.

**Results:**

Our search strategy generated 4,572 citations. The majority of citations were not relevant to the study objective. One study met inclusion criteria. In this retrospective study, morbidly obese youth with T2D were treated with a very low carbohydrate diet. This single study received a quality index score of < 11, indicating poor study quality and thus limiting confidence in the study's conclusions.

**Conclusions:**

There is no high quality evidence to suggest lifestyle modification improves either short- or long-term glycemic control in children and youth with T2D. Additional research is clearly warranted to define optimal lifestyle behaviour strategies for young people with T2D.

## Background

Globally, the prevalence of Type 2 Diabetes (T2D) in the pediatric population is increasing, most notably among 15 - 18 year olds [1-4]. At diagnosis, most boys and girls with T2D are overweight or obese, have a positive family history of T2D, are peri- or post-pubertal, and present with metabolic risk factors (e.g., dyslipidemia) [5]. With the early onset of this chronic condition and the associated co-morbidities, a life-long reduction in quality of life and premature mortality due to micro- and macro-vascular complications can be expected [6]. To address this health challenge resulting from pediatric T2D, effective and efficient management strategies are necessary.

Strong evidence supports the role of lifestyle modification to prevent (or at least delay) T2D in adults [7-11]. On this basis, many current clinical practice guidelines for adults with T2D recommend lifestyle modifications that include improving dietary quality as well as increasing the quantity and quality of physical activity to promote weight management and improve glycemic control [12-14]. Current treatment guidelines for children and youth with T2D do not differ from adult recommendations. Among children and youth who are asymptomatic (i.e., free of polyuria, polydipsia, or ketoacidosis) at diagnosis, intensive lifestyle counseling is recommended to achieve good glycemic control (i.e., HbA1c < 7.0% or fasting plasma glucose < 6.6 mmol/L) within 3 to 6 months [13-17]. If this clinical target is not achieved, initiation of metformin, an oral hypoglycemic agent, is recommended; in some cases, insulin therapy may also be necessary [16,18].

Although there is little data currently available on treatment patterns for children with T2D, it appears most boys and girls with T2D are treated pharmacologically [12]. It is not known whether this is a reflection of poor adherence to lifestyle modifications in children and youth or because clinicians' perceptions of and experiences with lifestyle recommendations and interventions are less effective for managing T2D in this population. To address this knowledge gap, we conducted a systematic review to evaluate the impact of lifestyle behavior modification on glycemic control among children and youth with clinically defined T2D.

## Methods

### Literature Search Strategy

A research librarian, with input from the research team, developed and implemented a comprehensive search strategy in selected high-yield electronic databases (EMBASE, CINAHL^®^, Cochrane Library, Medline^®^, PASCAL, PsycINFO^®^, and Sociological Abstracts) from their date of inception until October 2007. An updated search was completed in May 2009 (See Additional file1 for search terms). Relevant articles were also sought by searching the reference lists from articles retrieved for detailed review as well as related review articles published from January 2002 onward. Personal contact was established with content experts and authors of selected review articles to ensure relevant publications were not missed. No language restrictions were applied in this search strategy.

### Study Inclusion and Selection Criteria

We included all studies designed to evaluate the impact of lifestyle modification (diet and/or physical activity) on glycemic control (HbA1c) in children or youth with T2D. Lifestyle modification and glycemic control among children and youth with impaired glucose tolerance or impaired fasting glucose were not included. Similarly, the use of anti-diabetic drug therapies was not formally assessed. However, if a lifestyle modification group was included in any study design, the data were considered for inclusion. Studies were excluded if they did not include a comparison group or were not relevant to children and youth.

Two reviewers (STJ and MC) independently reviewed all abstracts and references. Studies were included if they met the following criteria: original research, participants ≤18 years of age with T2D, evaluated the effect of lifestyle modification (diet and/or physical activity) on glycemic control (HbA1c). Inter-observer agreement for study inclusion was high (κ = 0.92). Once the initial review was complete, a third investigator (GDCB) resolved any discrepancies by consensus.

### Quality Assessment

Assessment of the methodologic quality of included studies was completed using criteria from Downs and Black [19], which assessed study characteristics including internal and external validity, power, and reporting. A maximal quality index (QI) score was given to selected studies. A QI score >20 rated *good*, 11 to 20 rated *moderate *and <11 rated *poor *[19]. Two reviewers (STJ and MC) independently completed quality assessments of included studies. Any discrepancies were resolved through third party discussion (GDCB).

## Results

**Figure 1:**
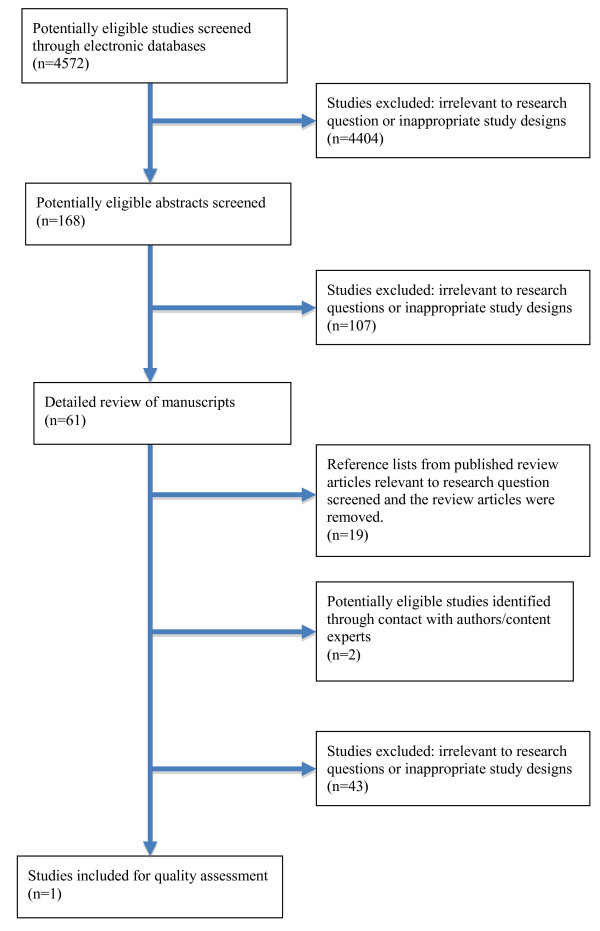
**Flow diagram of systematic search**.

Figure 1 shows the selection process for this systematic review. From the 4,572 publications identified by reviewing titles and abstracts, 61 manuscripts were selected for complete review. Of these manuscripts, 19 review articles were identified and removed. Additionally, seven cohort studies [20-26] and one case-study [27] were identified and reviewed because they were closely related to our research question, but did not fully match our inclusion criteria (Table 1). The cohort studies mainly described treatment trajectories, behavioral characteristics, disease management strategies, or quality of life among children and youth with T2D; however, they lacked a defined lifestyle intervention or comparator group. Although the identified case-study included a specific lifestyle intervention for one child with T2D, it was excluded based on the nature of the study design. One other study [28] described the evaluation of a community-based program that focused on food preparation skills, but did not include behavioral or clinical outcomes (Table 1). No additional studies were identified through our examination of study reference lists. Contact with experts in the field of pediatric T2D yielded two additional manuscripts, but upon review, these studies did not satisfy our inclusion criteria. In the end, one study (a retrospective, case-control design) met our inclusion criteria [29].

**Table 1:** Summary of publications that did not satisfy inclusion criteria, but were tangentially related to study research question regarding lifestyle intervention effects on HbA1c in children or youth with type 2 diabetes (T2D).

Study Authors	Primary Objective(s)	Research Methods	Intervention-Related Findings	Study Limitations
Zdravkovic *et al.*, 2004 [20]	To review the clinical experience of children and teens diagnosed with T2D at a pediatric hospital serving a large urban multi-ethnic population	Retrospective medical chart review (n = 41)	54% of patients were initially treated by lifestyle (diet and exercise); the minority (3%) did not require short-term intensification (i.e., medication) of therapy at some point since initial diagnosis to help achieve glycemia goals	Low quality study design; variable intervention elements (i.e., modality, duration, intensity); inconsistent outcome evaluation

Grinstein *et al.*, 2003 [21]	To report the presentation and 5-year treatment requirements of African-American and Caribbean-Hispanic adolescents with T2D followed at the Montefiore Medical Centre (Bronx, NY, USA)	Retrospective medical chart review (n = 83)	Most patients (63%) were prescribed oral medication (glipizide and/or metformin) and/or insulin. All patients were referred to a nutritionist for dietary counselling and recommendation of appropriate exercise intervention	Low quality study design; undefined lifestyle intervention elements (i.e., modality, duration, intensity); undocumented independent effects of lifestyle variables on glycemia

Zuhri-Yafi *et al.*, 2002 [22]	To study possible treatment modalities for type 2 diabetes in children and adolescents.	Retrospective medical chart review (n = 25); insulin the only initial treatment in 72% (n = 18); weight management strategies were taught and encouraged; insulin was withdrawn as euglycemia was achieved.	Mean change in HbA1c over 2 years: -2.9% for insulin users only and -2.3% for those treated with insulin and metformin, and -4.4.% for those treated by metformin alone. Few patents sustained any weight loss regardless of treatment.	Low quality study design; no description of diet and physical activity elements; inconsistent results with low statistical power to detect intervention effects.

Rothman *et al.*, 2008 [23]	To examine self-management behaviours and glycemic control among adolescents with T2D	Telephone survey + retrospective medical chart review (n = 103)	Minority (11.8%) used lifestyle changes exclusively as treatment. More than 80% of patients reported ≥75% medication compliance. More than 70% of patients reported exercising ≥2×/week; 68% reported viewing ≥2 hours of television daily. Patients reported frequent episodes of overeating, drinking sugar-sweetened beverages, and eating fast food. Many (37%) reported that 'following diet or exercise regime' was the 'hardest thing about having diabetes'	Data derived from cross-sectional, self-report survey and chart abstraction; non-specific intervention details queried

Reinehr *et al.*, 2008 [24]	To study the 2-year course of children and adolescents with T2D in general practice to present (1) the treatment modalities, (2) patient adherence, and (3) the occurrence of comorbidities.	Clinical data collected prospectively from 1995 to 2003 among 129 children and adolescents with type 2 diabetes from 62 specialized diabetes centers in Germany	Reduction in median HbA1c after 2 years; 60% of children dropped out of the study. Lifestyle intervention as sole treatment was usually not useful for achieving long-term metabolic control.	High number of cases lost to follow-up; poorly defined lifestyle intervention elements.

Shield *et al.*, 2009 [25]	To report the 1-year outcome for children newly diagnosed as having T2D across the UK.	Retrospective medical chart review (n = 73); follow-up occurred one year after incident cases were ascertained	Most common treatment at diagnosis was metformin (n = 34; 47%); lifestyle (diet and physical activity) was the initial treatment for a sub-group (n = 12; 17%); necessary lifestyle changes needed to positively affect metabolic health are not occurring; heterogeneity of treatment regimens appears relatively effective in achieving glycemic control.	Low quality study; inadequate detail regarding lifestyle intervention elements; no direct report of lifestyle behaviours.

Allan *et al.*, 2008 [26]	(1) To assess quality of life (QOL) in youth with type 2 diabetes, (2) to compare youth and parent-proxy perceptions of youth QOL, (3) to determine if youth QOL is associated with diabetes control, and (4) to determine if demographic and/or medical history is associated with youth QOL and/or diabetes control.	Cross-sectional survey and clinical data collection among First Nation youth aged 7-18 years and their parents at a regional diabetes program; 39% received lifestyle counselling alone with remainder receiving either insulin monotherapy or combination with oral hypoglycmeic agent; mean HbA1c 9.2 ± 2.9%	Youth reported higher scores in the generic and diabetes related domains compared to parents. Youth not taking diabetes medications reported higher QOL. QOL may be affected by specific demographic and clinical factors to reduce the psychosocial burden of their disease.	Low quality study; inadequate detail regarding lifestyle intervention elements.

Anderson & Dean (1990) [27]	To investigate the effect of a regulated food intake and daily exercise program on blood glucose levels, total glycosylated hemoglobin and weight status over a 3-year period.	Case study (n = 1); subject attended a month-long summer camp for three successive years.	Average HbA1c over 29 months was 18.4% with the lowest value of 16.9% occurring after summer camp. Controlled food intake and daily exercise improves glycemic control and in controlled environment only.	Low quality study design; small sample size limits generalizability; Glycemic target difficult to reach with intensive lifestyle intervention.

Nichol *et al.*, 2008 [28]	(1) To modify the Canadian Diabetes Association Pacific Area's *Cooking For Your Life! *Program for youth with T2D (or impaired glucose tolerance) and their families; (2) to evaluate program satisfaction	Pilot study (n = 15 adolescents; n = 21 family members); intervention included three 'hands-on' cooking classes + one grocery store tour	Three out of four sessions were attended by 86% of participants; 90% were 'mostly satisfied' or 'completely satisfied' with the program	Low quality study design; study not designed to impact glycemic control; study focussed on feasibility and process-related outcomes

HgbA1c - glycated hemoglobin; QOL - quality of life; T2D - type 2 diabetes.

In the study by Willi *et al.*, [29], the use of a very low carbohydrate diet in the treatment of T2D was found be an effective short-term therapy. However, this study was of poor methodological quality and its results should be interpreted with caution. The results are at high risk for bias because it was a convenience sample of hospital-based patients and was not a prospective design with random assignment to treatment and control. This study received a QI score of <11 (rated *poor*); additional details of this report are described in Table 2.

**Table 2:** Methods, design, and quality of studies for diet and physical activity in the treatment of pediatric type 2 diabetes mellitus.

Study Authors	Primary Objective(s)	Methods	Results	Authors' Conclusions
Willi *et al.*, 2004 (29)	To examine "the use of a ketogenic, very-low-calorie diet (VLCD) in the treatment of type 2 diabetes"	Retrospective case-control study, in a sample of 20 morbidly obese (body mass index (BMI) > 30 kg/m^2^) youth (14.5 ± 0.4 years old) with T2D. A control group (n = 15), matched by age, race, gender, BMI, T2D duration, HbA1c and pharmacotherapy who attended a hospital-based, outpatient pediatric diabetes clinic were included.	Participants who adhered to the VLCD for > 6 weeks (n = 15), had a 16% reduction in HbA1c (8.8 ± 0.6 to 7.4 ± 0.6, p < 0.005) after 8 weeks; adherence (total days) was associated with a greater reduction in HbA1c (r = -0.57, p < 0.01). In this same group, a reduction in BMI (43.5 ± 1.8 kg/m^2 ^to 39.3 ± 1.7 kg/m^2^, p < 0.0001) was observed after 8 weeks. At two years follow-up, glycemic control had returned to baseline in the VLCD group. Among those who consumed the VLCD diet for > 6 weeks (number not specified), BMI was significantly lower compared to baseline values within the treatment vs. control group (absolute HgbA1c values and statistical comparison data not provided). A sustained improvement in BMI in the VLCD group at 2 years follow-up compared to baseline assessment (41.2 ± 2.1 vs. 44.2 ± 2.3 vs. 41.2 ±21. kg/m^2^, p < 0.05) and compared to the control group, who had a 3.7% increase in BMI from baseline.	"The ketogenic VLCD is an effective short-term, and possibly long-term, therapy for pediatric patients with type 2 diabetes". (QI score = 8 or poor) ^**†**^

^**† **^Quality index based on Downs and Black (19) for the assessment of the methodological quality of randomized and nonrandomized studies of health interventions;

VLCD - Very Low Carbohydrate Diet; BMI - Body Mass Index; HgbA1c - glycated hemoglobin; kcal - kilocalories

## Discussion

Current treatment guidelines state that children and youth with T2D should receive intensive lifestyle counseling to help them achieve target glycemia within 3 - 6 months following diagnosis [12-16]. Despite this recommendation, our review of the literature revealed only one study that targeted lifestyle modification (diet) as an approach for improving glycemic control in this population. To date, most published studies have either included adults exclusively or have been based on retrospective or cross-sectional cohort studies of boys and girls with insulin resistance, but without T2D. Our review did not reveal any high quality studies that included physical activity interventions to improve short- or long-term glycemic control in children and youth with T2D, nor did it uncover any studies that examined the influence of combining diet and physical activity in the treatment of pediatric T2D. While we identified many review articles concerning the management of T2D in this population, none offered new data regarding the efficacy or effectiveness of lifestyle management for glycemic control.

The lack of published studies of lifestyle management for pediatric T2D may reflect the relatively low prevalence of the disease. Despite some having described pediatric diabetes to be at 'epidemic levels' [30-32], others argue prevalence estimates are of modest concern, even among those populations believed to be at greater risk [33]. Nevertheless, current prevalence estimates of overweight and obesity are cause for concern with respect to the potential for developing T2D and suggest a need for evidence-based recommendations for those who have already been diagnosed and those at high risk.

The Diabetes Prevention Program [7] and other lifestyle interventions for adults at high risk for T2D [8,9] have provided a model for clinicians and researchers upon which to base the design, delivery, and evaluation of clinical trials for T2D management. Indeed, these studies have informed the design of a recently-launched T2D management trial, which includes a lifestyle component [34]. However, it is important to bear in mind that pediatric and adult populations rarely receive similar therapies in research or clinical settings due to distinct metabolic, physical, developmental, and cognitive differences. Moreover, within the context of pediatric behavior modification, consideration of the complex interrelationships between environmental factors (i.e., family, peers, school, media, built environment) must be taken into account. Therefore, caution must be exercised when generalizing the clinical findings from currently available adult data to the pediatric population.

Of additional importance is the selection of appropriate study outcomes among the pediatric population [35]. Although good glycemic control and healthy body weights are of clinical importance, the antecedents of these clinical outcomes may be more salient [36]. For example, parental interactions with their sons and daughters when making family lifestyle changes have a meaningful impact [37] to the extent that the style with which parents communicate with and set boundaries within their family has considerable influence on children's nutrition and physical activity behaviours [38]. In this regard, evaluating outcomes such as parenting style, self-efficacy, and motivation to change lifestyle behaviours can help to contextualize nutrition and physical activity behaviours as well as metabolic outcomes that are influenced by lifestyle. Moreover, the current evidence-base for weight management may not be a suitable proxy for programs for pediatric T2D since many of the contemporary studies of pediatric weight management have been carried out on pre-adolescent children from primarily Caucasian, middle socioeconomic families. The current cohort of pediatric T2D patients includes (primarily) less affluent families of minority ethnic/racial backgrounds as well as families living with generations of chronic disease and co-morbidities of diabetes for which the disparities in health outcomes are well known in the adult T2D population.

Children and youth with T2D are usually overweight or obese [17]. Current pediatric and adult literature provides good evidence for reducing energy intake and increasing energy expenditure to enable weight management and reduce the risk of T2D. In adults, however, weight loss is not always necessary to improve glycemic control [39]. Until more evidence is available, it remains unknown whether glycemia can be improved in children and youth with T2D independent of weight loss. However, factors that can impact the achievement and sustainability of healthy lifestyle changes are increasingly being characterized. For example, a comprehensive health assessment prior to intervention enrolment would enable the design of interventions that are tailored to the needs of individuals and families, an advancement that can optimize outcomes in sub-groups with similar features. Weight management interventions that customize treatment based on loss of control eating [40], melanocortin 4 receptor gene mutation [41], maternal mental health [42], and/or motivation [43] could maximize individual responsiveness to weight management therapies. This degree of sophistication represents a substantial improvement beyond traditional variables (i.e., age, gender, obesity status) that, to date, have determined study inclusion and intervention approaches. This would also provide a degree of intervention sophistication that moves beyond a 'one size fits all' for managing T2D.

Presently, the U.S. National Institute of Diabetes and Digestive and Kidney Diseases (NIDDK) is supporting a number of large-scale, multi-centre trials designed to prevent or treat T2D in children and youth under the collaborative titled Studies to Treat Or Prevent Pediatric Type 2 Diabetes (STOPP-T2D). This partnership focuses on treating adolescents already diagnosed with T2D [34] and on the primary prevention of T2D among middle-school aged youth [44]. Unfortunately, following the completion of the NIDDK sponsored trial (Treatment Options for type 2 Diabetes in Adolescents and Youth: TODAY) [34], the independent effects of the dietary and physical activity behavioural changes on glycemic control will remain unknown; the trial does not include an independent lifestyle modification arm. Nevertheless, trials such as these are urgently needed to inform clinical practice.

The strengths of this study include the systematic, comprehensive and unbiased approach. The results of our systematic review should however be viewed in light of several limitations. An intrinsic limitation of any systematic review is the potential for publication and selection bias. We acknowledge this methodological drawback and undertook manual searches and contacted recognized experts in pediatric endocrinology. This strategy did not yield any additional unpublished articles that satisfied our inclusion criteria, so it is unlikely that we missed any relevant articles.

## Conclusion

In summary, our systematic review indicated that no well-designed studies have evaluated the impact of lifestyle modification on glycemic control in children and youth with T2D. Numerous review articles have been published in this area, but contribute little to our evidence base. Randomized clinical trials must be performed to clearly establish the role of nutrition and physical activity interventions in managing pediatric T2D. These studies might also help to determine the optimal lifestyle treatment approaches for good glycemic control independent of pharmacologic therapy for the pediatric T2D population. We believe that research to examine lifestyle-based therapies, which consider both qualitative and quantitative aspects of nutrition and physical activity in boys and girls with T2D, should remain research and public health priorities.

## Abbreviations

BMI: body mass index; HbA1c: glycated hemoglobin; Kg: kilogram; L: liter; m^2^: metres squared; mmol: millimole; NIDDK: Institute of Diabetes and Digestive and Kidney Diseases; STOPP-T2D: Studies to Treat Or Prevent Pediatric Type 2 Diabetes; TODAY: Treatment Options for type 2 Diabetes in Adolescents and Youth; T2D:type 2 diabetes; VLCD: very-low-calorie diet; QI: quality index; QOL: quality of life

## Competing interests

The authors declare that they have no competing interests.

## Authors' contributions

STJ contributed to study design, data collection, abstraction and interpretation, drafted the first manuscript and made subsequent revisions. ASN conceived the study, made substantial contributions to the study design and made critical revisions of early manuscript versions. MC participated in data collection and data abstraction. JB developed the search strategy and conducted the literature search. TTKH, PWF and MMJ provided critical revisions to the manuscript and provided important intellectual contributions. GDCB conceived the study, helped to solidify the study design and interpretation of data, drafted critical revisions, and, as did all authors, approved the final version of the manuscript.

## Pre-publication history

The pre-publication history for this paper can be accessed here:

http://www.biomedcentral.com/1471-2431/10/97/prepub

## Supplementary Material

Additional file 1 **Appendix A: Search strategies**. Overview of systematic review search strategies. Click here for file
