# Supplementary material for: In search of quality evidence for lifestyle management and glycemic control in children and adolescents with type 2 diabetes: A systematic review
Source: BMC Pediatr. 2010 Dec 23;10:97. doi: 10.1186/1471-2431-10-97 (PMC3016367; doi:10.1186/1471-2431-10-97)
Supplement: Additional file 1 — Appendix A: Search strategies. Overview of systematic review search strategies. [file 1471-2431-10-97-S1.DOC]

**Appendix A: Search strategies**

**Medline (via OVID interface)**

1. exp Diabetes Mellitus, Type 2/

2. ((adult-onset or adult onset or ketosis-resistant or maturity-onset or non-insulin-dependent or non insulin dependent or noninsulin dependent or slow-onset or slow onset or stable or maturity onset or maturity-onset or type II or type 2) adj3 diabetes mellitus).mp. [mp=title, original title, abstract, name of substance word, subject heading word]

3. (Niddm or mody).mp. [mp=title, original title, abstract, name of substance word, subject heading word]

4. 1 or 2 or 3

5. limit 4 to "all child (0 to 18 years)"

6. exp Metabolic Syndrome X/

7. (metabolic adj2 syndrome$).mp. [mp=title, original title, abstract, name of substance word, subject heading word]

8. ((insulin resistan$ or dysmetabolic or reaven) adj3 syndrome$).mp. [mp=title, original title, abstract, name of substance word, subject heading word]

9. exp Insulin Resistance/

10. exp Abdominal Fat/

11. exp Obesity/

12. exp Hyperglycemia/

13. Hypertension/

14. (prothrombo$ or proinflamma$).mp. [mp=title, original title, abstract, name of substance word, subject heading word]

15. 6 or 7 or 8 or 9 or 10 or 11 or 12 or 13 or 14

16. limit 15 to "all child (0 to 18 years)"

17. 5 or 16

18. exp Life Style/

19. exp Health Behavior/

20. diet/ or diabetic diet/ or diet, carbohydrate-restricted/ or diet, fat-restricted/ or diet, mediterranean/ or diet, reducing/

21. exp Diet Therapy/

22. dh.fs.

23. Exercise Therapy/

24. exercise/ or exertion/

25. (lifestyle or environmental$ or community or exercis$ or diet$ or nutrition$ or food$ or activity or activities or meals or mealtime$ or sport$).mp. [mp=title, original title, abstract, name of substance word, subject heading word]

26. exp Sports/

27. 18 or 19 or 20 or 21 or 22 or 23 or 24 or 25 or 26

28. 17 and 27

29. exp Blood Glucose/

30. exp Body Composition/

31. exp Body Mass Index/

32. exp Weight Loss/

33. exp Blood Pressure/

34. cholesterol/ or cholesterol, hdl/ or cholesterol, ldl/ or cholesterol, vldl/

35. exp Triglycerides/

36. 29 or 30 or 31 or 32 or 33 or 34 or 35

37. 28 and 36

Filtered for controlled trials (based on Cochrane Highly Sensitive Search filter for randomized controlled trials):

38. randomized controlled trial.pt.

39. controlled clinical trial.pt.

40. meta analysis.pt.

41. exp Randomized Controlled Trials/

42. exp Random Allocation/

43. exp Double-Blind Method/

44. exp Single-Blind Method/

45. exp Meta-Analysis/

46. ((singl$ or doubl$ or tripl$ or trebl$) adj2 (blind$ or mask$)).ti,ab.

47. random$.ti,ab.

48. (systematic adj2 (review$ or overview$)).ti,ab.

49. 38 or 39 or 40 or 41 or 42 or 43 or 44 or 45 or 46 or 47 or 48

50. limit 49 to animals

51. limit 49 to (humans and animals)

52. 50 not 51

53. 49 not 52

54. clinical trial.pt.

55. exp Clinical Trials/

56. (clin$ adj2 trial$).ti,ab.

57. exp Placebos/

58. placebo$.ti,ab.

59. exp Research Design/

60. 54 or 55 or 56 or 57 or 58 or 59

61. limit 60 to animals

62. limit 60 to (humans and animals)

63. 61 not 62

64. 60 not 63

65. comparative study.pt.

66. exp Evaluation Studies/

67. exp Follow-Up Studies/

68. exp Prospective Studies/

69. (control$ or prospective$).mp. or volunteer$.ti,ab. [mp=title, original title, abstract, name of substance word, subject heading word]

70. compar$.ti,ab.

71. 65 or 66 or 67 or 68 or 69 or 70

72. limit 71 to animals

73. limit 71 to (humans and animals)

74. 72 not 73

75. 71 not 74

76. 53 or 64 or 75

77. 37 and 76

78. 38 or 39

79. 77 and 78

80. 4 or 7 or 8

81. limit 80 to "all child (0 to 18 years)"

82. 81 and 27

83. 9 or 10 or 11 or 12 or 13 or 14 or 36

84. 82 and 83

85. 84 and 76

86. 84 and 78

87. 38 or 39 or 54 or 65

88. 84 and 87

89. 37 and 87

**EMBASE (via Ovid interface)**

1. exp Diabetes Mellitus, Type 2/

2. ((adult-onset or adult onset or ketosis-resistant or maturity-onset or non-insulin-dependent or non insulin dependent or noninsulin dependent or slow-onset or slow onset or stable or maturity onset or maturity-onset or type II or type 2) adj3 diabetes mellitus).mp. [mp=title, abstract, subject headings, heading word, drug trade name, original title, device manufacturer, drug manufacturer name]

3. (Niddm or mody).mp. [mp=title, abstract, subject headings, heading word, drug trade name, original title, device manufacturer, drug manufacturer name]

4. 1 or 2 or 3

5. exp Metabolic Syndrome X/

6. (metabolic adj2 syndrome$).mp. [mp=title, abstract, subject headings, heading word, drug trade name, original title, device manufacturer, drug manufacturer name]

7. ((insulin resistan$ or dysmetabolic or reaven) adj3 syndrome$).mp. [mp=title, abstract, subject headings, heading word, drug trade name, original title, device manufacturer, drug manufacturer name]

8. exp Insulin Resistance/

9. exp Abdominal Fat/

10. exp Obesity/

11. exp Hyperglycemia/

12. Hypertension/

13. (prothrombo$ or proinflamma$).mp. [mp=title, abstract, subject headings, heading word, drug trade name, original title, device manufacturer, drug manufacturer name]

14. 5 or 6 or 7 or 8 or 9 or 10 or 11 or 12 or 13

15. exp Life Style/

16. exp Health Behavior/

17. diet/ or diabetic diet/ or diet, carbohydrate-restricted/ or diet, fat-restricted/ or diet, mediterranean/ or diet, reducing/

18. exp Diet Therapy/

19. dh.fs.

20. Exercise Therapy/

21. exercise/ or exertion/

22. (lifestyle or environmental$ or community or exercis$ or diet$ or nutrition$ or food$ or activity or activities or meals or mealtime$ or sport$).mp. [mp=title, abstract, subject headings, heading word, drug trade name, original title, device manufacturer, drug manufacturer name]

23. exp Sports/

24. 15 or 16 or 17 or 18 or 19 or 20 or 21 or 22 or 23

25. exp Blood Glucose/

26. exp Body Composition/

27. exp Body Mass Index/

28. exp Weight Loss/

29. exp Blood Pressure/

30. cholesterol/ or cholesterol, hdl/ or cholesterol, ldl/ or cholesterol, vldl/

31. exp Triglycerides/

32. 25 or 26 or 27 or 28 or 29 or 30 or 31

33. 4 or 14

34. 24 and 32 and 33

35. limit 34 to (preschool child <1 to 6 years> or school child <7 to 12 years> or adolescent <13 to 17 years>)

36. limit 34 to (adult <18 to 64 years> or aged <65+ years>)

37. 35 not 36

Filtered for randomized controlled trials and other controlled clinical trials:

38. Randomized Controlled Trial/

39. exp RANDOMIZATION/

40. Double Blind Procedure/

41. Single Blind Procedure/

42. Meta Analysis/

43. exp "Systematic Review"/

44. ((singl$ or doubl$ or tripl$ or trebl$) adj2 (blind$ or mask$)).ti,ab.

45. random$.ti,ab.

46. (systematic adj2 (review$ or overview$)).ti,ab.

47. 38 or 39 or 40 or 41 or 42 or 43 or 44 or 45 or 46

48. exp ANIMAL/

49. Human/

50. 48 and 49

51. 48 not 50

52. 47 not 51

53. Clinical Trial/

54. (clin$ adj2 trial$).ti,ab.

55. exp PLACEBO/

56. placebo$.ti,ab.

57. 53 or 54 or 55 or 56

58. 57 not 51

59. exp Comparative Study/

60. exp EVALUATION/

61. exp Follow Up/

62. exp Prospective Study/

63. (control$ or prospective$).mp. or volunteer$.ti,ab. [mp=title, abstract, subject headings, heading word, drug trade name, original title, device manufacturer, drug manufacturer name]

64. compar$.ti,ab.

65. 59 or 60 or 61 or 62 or 63 or 64

66. 65 not 51

67. 52 or 58 or 66

68. exp Comparative Study/

69. exp Prospective Study/ or exp Cohort Analysis/

70. exp Longitudinal Study/

71. exp Follow Up/

72. 67 or 68 or 69 or 70 or 71

73. 37 and 72

**PASCAL (via Ovid)**

1. ((adult-onset or adult onset or ketosis-resistant or maturity-onset or non-insulin-dependent or non insulin dependent or noninsulin dependent or slow-onset or slow onset or stable or maturity onset or maturity-onset or type II or type 2) adj3 diabetes mellitus).mp. [mp=title, abstract, heading word, table of contents, key concepts]

2. (Niddm or mody).mp. [mp=title, abstract, heading word, table of contents, key concepts]

3. (metabolic adj2 syndrome$).mp. [mp=title, abstract, heading word, table of contents, key concepts]

4. ((insulin resistan$ or dysmetabolic or reaven) adj3 syndrome$).mp. [mp=title, abstract, heading word, table of contents, key concepts]

5. (prothrombo$ or proinflamma$).mp. [mp=title, abstract, heading word, table of contents, key concepts]

6. (lifestyle or environmental$ or community or exercis$ or diet$ or nutrition$ or food$ or activity or activities or meals or mealtime$ or sport$).mp. [mp=title, abstract, heading word, table of contents, key concepts]

7. 1 or 2 or 3 or 4

8. 6 and 7

9. (adolescen$ or teen$ or child$).mp. and 8 [mp=title, abstract, heading word, table of contents, key concepts]

**All other databases (CINAHL, Sociological Abstracts, PsycInfo, Scopus) searched with textword strategy**:

1. ((“adult-onset” OR “adult onset” OR “ketosis-resistant” OR “maturity-onset” OR “non-insulin-dependent” OR “non insulin dependent” OR “noninsulin dependent” OR “slow-onset” OR “slow onset” OR stable OR “maturity onset” OR “maturity-onset” OR “type II” OR “type 2”) AND diabetes)) OR (Niddm or mody) OR “metabolic syndrome” OR ((“insulin resistant”OR “insulin resistance” OR dysmetabolic or reaven) AND syndrome*)
2. (lifestyle or environmental* or community or exercise* or diet* or nutrition* or food* or activity or activities or meals or mealtime* or sport*)
3. child* OR adolescent* OR teen*
4. 1 AND 2 AND 3
